# Intravitreal aflibercept 8 mg in patients with neovascular age-related macular degeneration from China: 48-week results from the PULSAR trial

**DOI:** 10.1186/s40942-026-00876-5

**Published:** 2026-06-26

**Authors:** Xiaodong Sun, Hong Dai, Youxin Chen, Xufang Sun, Liming Tao, Jun Xiao, Mei Han, Mingwei Zhao, Li Wang, Andrea Schulze, Ursula Maria Schmidt-Ott, Min Zhao, Xin Zhang, Zhaohong Wang, Sergio Leal, Wenbin Wei

**Affiliations:** 1 Shanghai General Hospital, Shanghai Jiao Tong University School of Medicine, Shanghai, China; 2 Beijing Hospital, Beijing, China; 3 Peking Union Medical College Hospital, Beijing, China; 4 Tongji Hospital, Tongji Medical College, Huazhong University of Science and Technology, Wuhan, China; 5 The Second Affiliated Hospital of Anhui Medical University, Anhui, China; 6 The Second Affiliated Hospital of Jilin University, Changchun, China; 7 Tianjin Eye Hospital, Tianjin, China; 8 Peking University People’s Hospital, Beijing, China; 9 Bayer Healthcare Company Limited, Beijing, China; 10 Bayer AG, Berlin, Germany; 11 Bayer Consumer Care AG, Basel, Switzerland; 12 Beijing Tongren Hospital, Capital Medical University, Beijing, China

**Keywords:** Aflibercept, Neovascular age-related macular degeneration, Anti-VEGF agent, Vascular endothelial growth factor

## Abstract

**Background:**

PULSAR (NCT04423718) was a global, phase 3, randomized, double-masked, non-inferiority study of adults with neovascular age-related macular degeneration (nAMD). Patients were randomized 1:1:1 to receive aflibercept 8 mg every 12 (8q12), or 16 weeks (8q16), or aflibercept 2 mg every 8 weeks (2q8), following 3 initial monthly doses. This subgroup analysis investigated the efficacy and safety of aflibercept 8 mg vs. aflibercept 2 mg in patients from China with nAMD from the PULSAR trial.

**Methods:**

This exploratory analysis evaluated the change from baseline in best-corrected visual acuity (BCVA), central retinal thickness (CRT), durability, and safety outcomes through week 48 in patients from China. All results were descriptive in nature.

**Results:**

Least squares (LS) mean (95% confidence interval [CI]) change from baseline in BCVA at week 48 was + 13.2 (+9.0, +17.4), +9.7 (+5.8, +13.7), and + 10.0 (+6.9, +13.1) letters for patients from China in the 8q12 (*n* = 31), 8q16 (*n* = 31), and 2q8 (*n *= 39) groups, respectively. LS mean (95% CI) change from baseline in CRT (µm) at week 48 was –167 (–183, −151), −166 (–195, −137), and −180 (–195, −165) for patients in the 8q12, 8q16, and 2q8 groups, respectively. Randomized dosing intervals were maintained by 88.5% and 73.1% of patients in the 8q12 and 8q16 groups, respectively. The incidence of ocular treatment-emergent adverse events was similar across treatment groups.

**Conclusions:**

In patients with nAMD from China, aflibercept 8 mg administered over extended dosing intervals demonstrated efficacy and a safety profile consistent with that observed with aflibercept 2 mg, in line with the overall PULSAR cohort.

**Trial registration:**

ClinicalTrials.gov, TRN NCT04423718. Registration date 8 June 2020.

**Supplementary Information:**

The online version contains supplementary material available at https://doi.org/10.1186/s40942-026-00876-5.

## Background

Neovascular age-related macular degeneration (nAMD) is one of the leading causes of vision loss worldwide [1, 2]. In China, more than 8 million people are projected to be affected by nAMD by 2050 and the prevalence of this condition will likely increase in the future due to a rapidly increasing elderly population [2–4]. Intravitreal anti-vascular endothelial growth factor (VEGF) agents are the standard of care for nAMD in China, as well as the Asia-Pacific region [5, 6]. Findings from a global real-world study, which included China, have demonstrated suboptimal functional outcomes with anti-VEGF (ranibizumab) treatment compared with those seen in pivotal clinical trials, mostly related to receiving fewer injections than recommended in the label [7]. This may, in turn, be attributed to undertreatment, non-adherence, and the burden associated with disease management, including the need for regular clinic visits for monitoring and injections [8]. The development of intravitreal anti-VEGF agents that can be administered with extended dosing intervals may alleviate patient burden and promote adherence to treatment [9–11], leading to improvement in patient outcomes. PULSAR (NCT04423718) was a global, randomized, double-masked, active-controlled, 96-week, phase 3 trial that evaluated the efficacy and safety of aflibercept 8 mg vs. aflibercept 2 mg [12]. Adults with nAMD were randomized in a 1:1:1 ratio to receive aflibercept 8 mg every 12 weeks (8q12), aflibercept 8 mg every 16 weeks (8q16), or aflibercept 2 mg every 8 weeks (2q8), each after 3 initial monthly doses. At week 48, the 8q12 and 8q16 groups showed non-inferiority in best-corrected visual acuity (BCVA) gains at a 4-letter margin compared with the 2q8 group. Most patients receiving aflibercept 8 mg maintained extended dosing intervals (83% overall maintained ≥ 12-week intervals, and 77% of those in the 8q16 group maintained 16-week intervals). Significantly more patients receiving aflibercept 8 mg had no retinal fluid in the center subfield at week 16 compared with those receiving aflibercept 2 mg, and the rates of treatment-emergent adverse events were similar across treatment groups [12]. To date, aflibercept 8 mg has been approved for the treatment of nAMD across Europe, in the United States, and Japan, and most recently, in China [13–16].

This subgroup analysis of the PULSAR trial describes the efficacy, safety, and durability of aflibercept 8 mg vs. aflibercept 2 mg in patients from China though 48 weeks of treatment, which will help to inform the therapeutic benefits of aflibercept 8 mg in Chinese patients with nAMD.

## Methods

### Study design

Study details for PULSAR (NCT04423718) have previously been published [12]. In brief, PULSAR was a randomized, double-masked, non-inferiority, phase 3 study conducted in 223 clinical sites in 27 countries in accordance with the Declaration of Helsinki, International Council for Harmonization Good Clinical Practice Guidelines, and any applicable local laws and regulations. Prior to the initiation of PULSAR, the study protocol was approved by relevant institutional review boards and independent ethics committees.

Patients with nAMD aged ≥ 50 years were eligible for enrollment, and one eye per patient was designated as the study eye. Eligible patients were randomly assigned to the 8q12, 8q16, and 2q8 treatment groups in a 1:1:1 ratio through an interactive web response system. Patients received 3 initial monthly doses prior to continuing treatment per their randomly assigned dosing schedule. Patients received either an aflibercept or sham injection (to maintain masking) in accordance with their assigned treatment group and eligibility for dose regimen modification (DRM). The prespecified disease activity criteria for DRM, denoting disease activity, were > 5-letter loss in BCVA from week 12, and either a> 25 μm increase in central retinal thickness (CRT) or new foveal hemorrhage or neovascularization. Patients in the 8q12 or 8q16 groups who met the DRM criteria at week 16 or week 20, had their dosing interval shortened to every 8 weeks (q8). Patients in the 8q12 and 8q16 groups who did not meet DRM criteria at week 16 or 20 but did at an injection visit from week 24 onwards had their dosing intervals shortened by 4 weeks. Patients in the 2q8 group continued with q8 dosing through week 48 [12].

The minimum dosing interval for all patients was every 8 weeks, and it could not be extended within the first year of treatment. Patients were monitored every 4 weeks, and sham injections were not given at the monitoring visit at week 12.

### Outcomes

This analysis evaluated functional, anatomic, durability, and safety outcomes in two subgroups: patients enrolled from China (China subgroup) and patients enrolled from outside China (non-China subgroup). Efficacy endpoints included the change from baseline in BCVA (Early Treatment Diabetic Retinopathy Study [ETDRS] letter score) at week 48, change in CRT (measured from the inner limiting membrane to the retinal pigment epithelium) at week 48, and the proportion of patients with no retinal fluid (referring to the abnormal accumulation of fluid within the retinal layers [intraretinal] or outside the foveal center and extending throughout the entire macular region [subretinal]) [17] in the center subfield, at weeks 16 and 48. Durability was assessed in terms of the proportion of patients in the 8q12 and 8q16 groups who maintained their 12- or 16-week dosing intervals through week 48, respectively. Safety endpoints included the incidence of ocular and non-ocular treatment-emergent adverse events (TEAEs) and serious TEAEs through week 48. Ocular and non-ocular TEAEs were reported and coded using the Medical Dictionary for Regulatory Activities version 25.0.

### Statistical analysis

In this exploratory subgroup analysis, all efficacy endpoints were reported for the full analysis set, which included all patients who were randomly assigned to treatment and received ≥ 1 dose of study intervention. Patients were analyzed by their treatment group at randomization. Safety analyses were performed in the safety analysis set, which included all randomized patients who received ≥ 1 dose of study intervention, and patients were analyzed as treated. No confirmatory statistical analyses were performed between China and non-China subgroups, as PULSAR was not designed or powered to detect differences between treatment groups within subgroups.

The primary efficacy endpoints for this subgroup analysis were assessed using the same methodology as in the original PULSAR trial for the overall population [12]. Briefly, the least squares (LS) mean change from baseline to week 48 in BCVA, and CRT, was calculated using a mixed model for repeated measures (MMRM) method that included baseline values (BCVA and CRT, respectively) as a covariate, as previously described [12]. Additional information can be found in the Supplementary Material 1.

For binary efficacy endpoints (the proportion of patients without retinal fluid in the center subfield at weeks 16 and 48), the last observation carried forward method was used, imputing missing values for patients who had at least 1 post-baseline value. The durability endpoints (the proportion of patients who maintained their dosing intervals) and treatment exposure (number of injections) were analyzed based on the subset of patients who completed 48 weeks. The safety endpoints were summarized descriptively. Statistical evaluation was performed using the software package SAS version 9.4 or higher. No formal hypothesis testing between treatment groups or regions was performed. All analyses were descriptive and hypothesis-generating only.

## Results

### Patients

In the PULSAR trial, a total of 1009 patients received aflibercept treatment. Of these patients, 101 patients were included in the China subgroup (8q12: *n* = 31; 8q16: *n* = 31; 2q8: *n* = 39) and 908 in the non-China subgroup (8q12: *n* = 304; 8q16: *n* = 307; 2q8: *n* = 297) (Table 1, Supplementary Fig. 1). In the China subgroup, 85 patients (84.2%) completed treatment through week 48, and 852 patients (93.8%) in the non-China subgroup completed treatment through week 48 (Supplementary Fig. 1).

Patient demographics and baseline clinical characteristics, including BCVA, were generally consistent across treatment groups within the China and non-China subgroups (Table 1). Overall, 29.0%–35.5% and 55.7%–58.9% of patients in the China and non-China subgroups, respectively, were female. The proportion of patients with predominantly classic choroidal neovascularization (CNV) ranged from 16.1% to 17.9% in the China subgroup and 20.2%–21.7% in the non-China subgroup, BCVA ranged from 52.8 to 57.8 letters in the China subgroup and 59.1–60.6 letters in the non-China subgroup, and CRT ranged from 370 to 397 μm in the China subgroup and 363–371 μm in the non-China subgroup. Among those patients who underwent optional indocyanine green angiography (ICGA), polypoidal choroidal vasculopathy (PCV) diagnosis was confirmed in 55.6%, 42.1%, and 60.7% of patients in the 8q12, 8q16, and 2q8 groups, respectively, in the China subgroup, and 42.5%, 48.5%, and 46.8% of patients, respectively, in the non-China subgroup.

Patients who completed 48 weeks of treatment in the 8q12, 8q16, and 2q8 groups in the China subgroup received a mean of 5.5, 4.7, and 6.6 aflibercept injections, respectively, while those in the non-China subgroup received a mean of 6.0, 5.1, and 6.8 injections, respectively.

**Table 1:** Baseline demographics and disease characteristics (study eye) of the China and non-China subgroups in PULSAR

	China				Non-China			
	Aflibercept 2q8 (*n* = 39)	Aflibercept 8q12 (*n* = 31)	Aflibercept 8q16 (*n* = 31)	Combined aflibercept 8 mg (*n* = 62)	Aflibercept 2q8 (*n* = 297)	Aflibercept 8q12 (*n* = 304)	Aflibercept 8q16 (*n* = 307)	Combined aflibercept 8 mg (*n* = 611)
Age, years	68.5 (8.7)	69.1 (5.9)	68.7 (6.6)	68.9 (6.2)	74.9 (8.6)	75.3 (7.9)	75.0 (8.5)	75.2 (8.2)
Sex, n (%)								
Female	13 (33.3)	11 (35.5)	9 (29.0)	20 (32.3)	175 (58.9)	171 (56.3)	171 (55.7)	342 (56.0)
Male	26 (66.7)	20 (64.5)	22 (71.0)	42 (67.7)	122 (41.1)	133 (43.8)	136 (44.3)	269 (44.0)
Race, n (%)								
Asian	39 (100)	31 (100)	31 (100)	62 (100)	44 (14.8)	43 (14.1)	46 (15.0)	89 (14.6)
Black or African American	0	0	0	0	2 (0.7)	2 (0.7)	0	2 (0.3)
Multiple	0	0	0	0	0	1 (0.3)	0	1 (0.2)
Not reported	0	0	0	0	2 (0.7)	2 (0.7)	1 (0.3)	3 (0.5)
White	0	0	0	0	249 (83.8)	256 (84.2)	260 (84.7)	516 (84.5)
BCVA, ETDRS letter score	57.8 (14.1)	52.8 (15.1)	56.3 (12.3)	54.6 (13.8)	59.1 (14.0)	60.6 (13.0)	60.4 (12.4)	60.5 (12.7)
CRT, µm	397 (167)	372 (143)	370 (173)	371 (158)	363 (128)	370 (122)	371 (128)	370 (125)
CNV size, mm^2^	6.1 (5.3)	5.8 (4.8)	6.2 (4.0)	6.0 (4.4)	6.4 (5.0)	6.0 (4.9)	6.6 (5.7)	6.3 (5.3)
Type of CNV, n (%)								
Predominantly classic	7 (17.9)	5 (16.1)	5 (16.1)	10 (16.1)	64 (21.5)	66 (21.7)	62 (20.2)	128 (20.9)
Minimally classic	11 (28.2)	9 (29.0)	9 (29.0)	18 (29.0)	50 (16.8)	47 (15.5)	59 (19.2)	106 (17.3)
Occult only	20 (51.3)	15 (48.4)	14 (45.2)	29 (46.8)	172 (57.9)	182 (59.9)	172 (56.0)	354 (57.9)
PCV presence (ICGA confirmed), n/N (%)^a^	17/28 (60.7)	10/18 (55.6)	8/19 (42.1)	18/37 (48.6)	37/79 (46.8)	34/80 (42.5)	33/68 (48.5)	67/148 (45.3)

Data are mean (SD) unless otherwise noted.

^a^ICGA was not performed for all patients; as such, the actual numbers of patients with PCV may be different than reported here. Continuous data are summarized by mean (SD) and categorical data by frequency counts and percentages. CRT was measured from the inner limiting membrane to the retinal pigment epithelium. CNV reported as per reading center.

2q8, aflibercept 2 mg every 8 weeks; 8q12, aflibercept 8 mg every 12 weeks; 8q16, aflibercept 8 mg every 16 weeks; BCVA, best-corrected visual acuity; CNV, choroidal neovascularization; CRT, central retinal thickness; EDTRS, Early Treatment Diabetic Retinopathy Study; ICGA, indocyanine green angiography; N, number of patients with ICGA assessment; PCV, polypoidal choroidal vasculopathy; SD, standard deviation.

### Functional outcomes

**Fig. 1:**
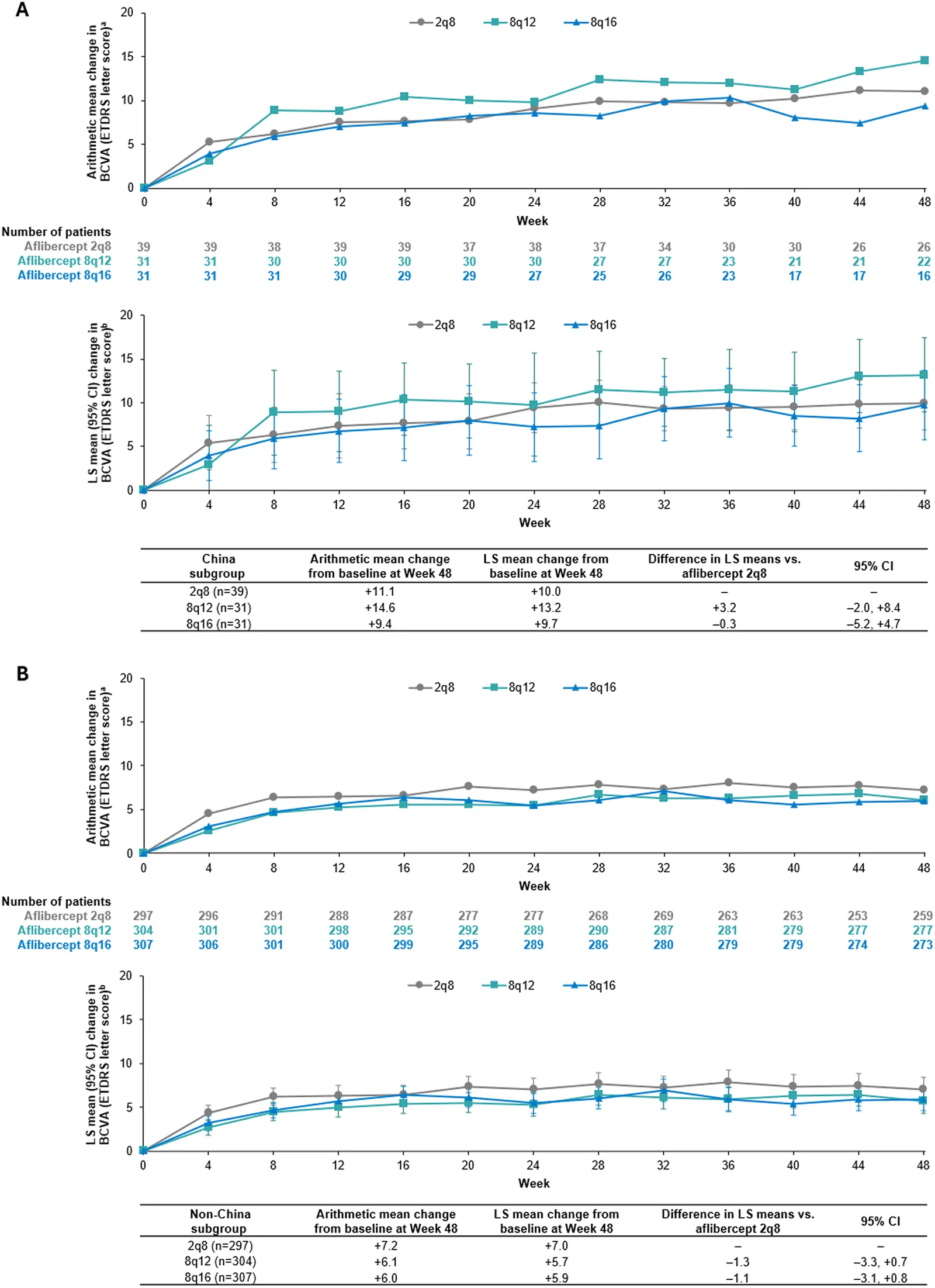
Arithmetic mean change (top panel) and LS mean change (bottom panel) in BCVA through week 48 in the China (**A**) and non-China (**B**) subgroups. Full analysis set. ^a^Arithmetic mean changes based on OC prior to ICE. The number of patients included in the analysis, excluding patients with relevant ICE, are provided for each timepoint; ^b^LS mean data were generated using MMRM, with baseline BCVA as a covariate, treatment group (i.e., aflibercept 2q8, 8q12, or 8q16), visit, and stratification variables (i.e., geographical region [China vs. rest of the world] and baseline BCVA [< 60 vs. ≥ 60 letters]) as fixed factors, and terms for the interaction between baseline and visit and the interaction between treatment and visit. Missing data were covered by the MMRM. 2q8, aflibercept 2 mg every 8 weeks; 8q12, aflibercept 8 mg every 12 weeks; 8q16, aflibercept 8 mg every 16 weeks. BCVA, best-corrected visual acuity; CI, confidence interval; ETDRS, Early Treatment Diabetic Retinopathy Study; ICE, intercurrent events; LS, least squares; MMRM, mixed model for repeated measures; OC, observed cases

Improvements in BCVA were observed in the 8q12, 8q16, and 2q8 groups in both the China and non-China subgroups at week 48 (Fig. 1). In the China subgroup, mean (standard deviation [SD]) change from baseline in BCVA at week 48 was + 14.6 (12.6), + 9.4 (12.1), and + 11.1 (9.6) letters for patients in the 8q12, 8q16, and 2q8 groups, respectively. The corresponding LS mean (95% CI) change from baseline was +13.2 (+9.0, +17.4), +9.7 (+5.8, +13.7), and +10.0 (+6.9, +13.1) letters for those in the 8q12, 8q16, and 2q8 groups, respectively. The difference in LS mean (95% CI) change from baseline was +3.2 (–2.0, +8.4) between the 8q12 and 2q8 groups and −0.3 (–5.2, +4.7) between the 8q16 and 2q8 groups. In the non-China subgroup, the LS mean (95% CI) change from baseline was +5.7 (+4.3, +7.1), +5.9 (+4.6, +7.2), and +7.0 (+5.6, +8.4) letters for patients in the 8q12, 8q16, and 2q8 groups, respectively. The difference in LS mean (95% CI) change from baseline was −1.3 (–3.3, +0.7) between the 8q12 and 2q8 groups and –1.1 (–3.1, +0.8) between the 8q16 and 2q8 groups.

### Anatomic outcomes

**Fig. 2:**
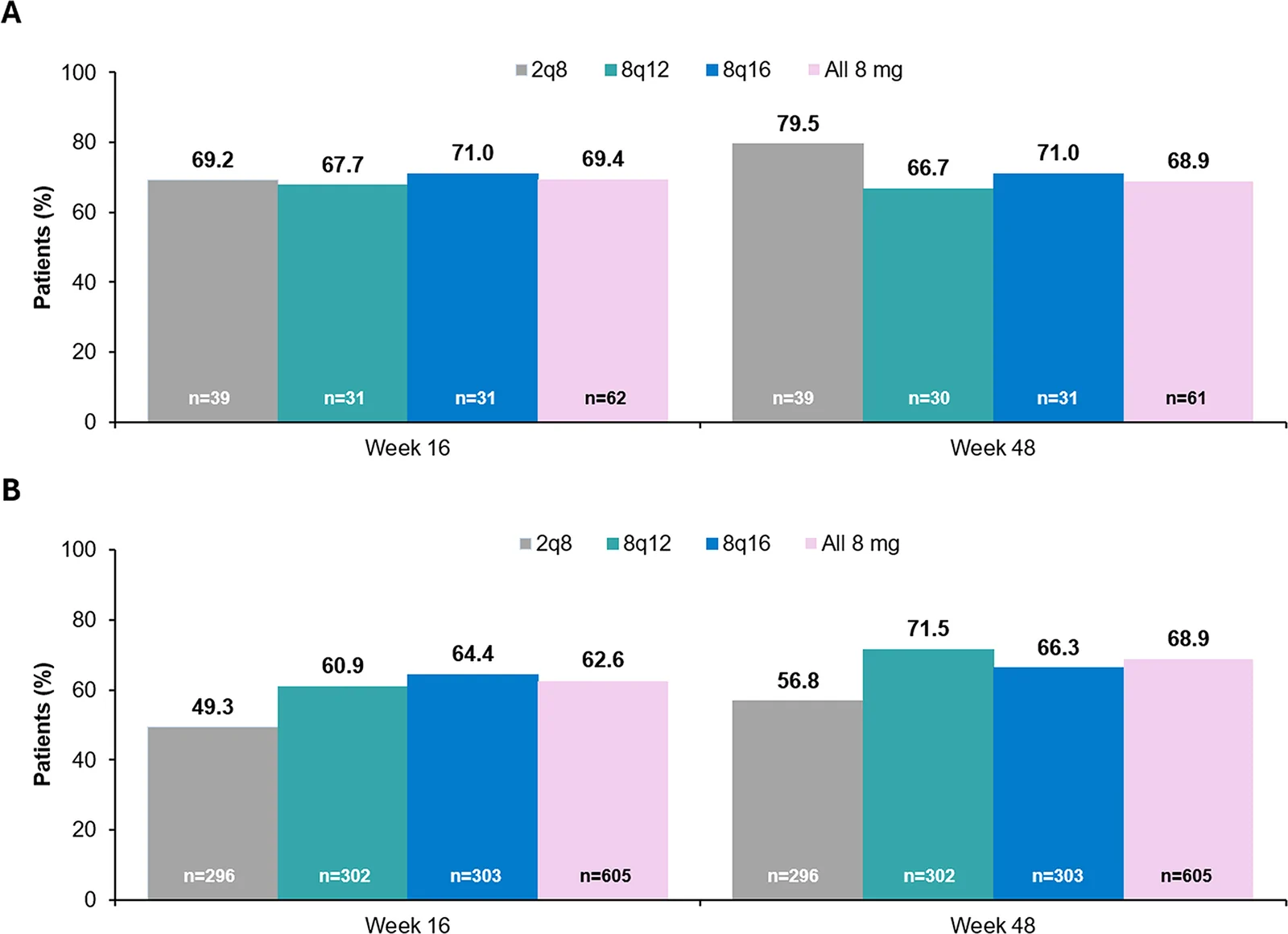
Proportion of patients without retinal fluid in the center subfield at weeks 16 and 48 in the China (**A**) and non-China (**B**) subgroups. Full analysis set. Last observation carried forward. Last available observed value prior to ICE was used to impute missing data. 2q8, aflibercept 2 mg every 8 weeks; 8q12, aflibercept 8 mg every 12 weeks; 8q16, aflibercept 8 mg every 16 weeks; ICE, intercurrent events

In the China subgroup, 69.4% of patients treated with aflibercept 8 mg had no retinal fluid (subretinal or intraretinal) present in the center subfield at week 16 (8 weeks following 3 initial monthly injections) compared with 69.2% in the 2q8 group (Fig. 2A). In the non-China subgroup, 62.6% of patients receiving aflibercept 8 mg had no retinal fluid in the center subfield at week 16 compared with 49.3% in the 2q8 group (Fig. 2B). Fluid control achieved at week 16 was sustained through week 48 in all treatment groups in both subgroups.

**Fig. 3:**
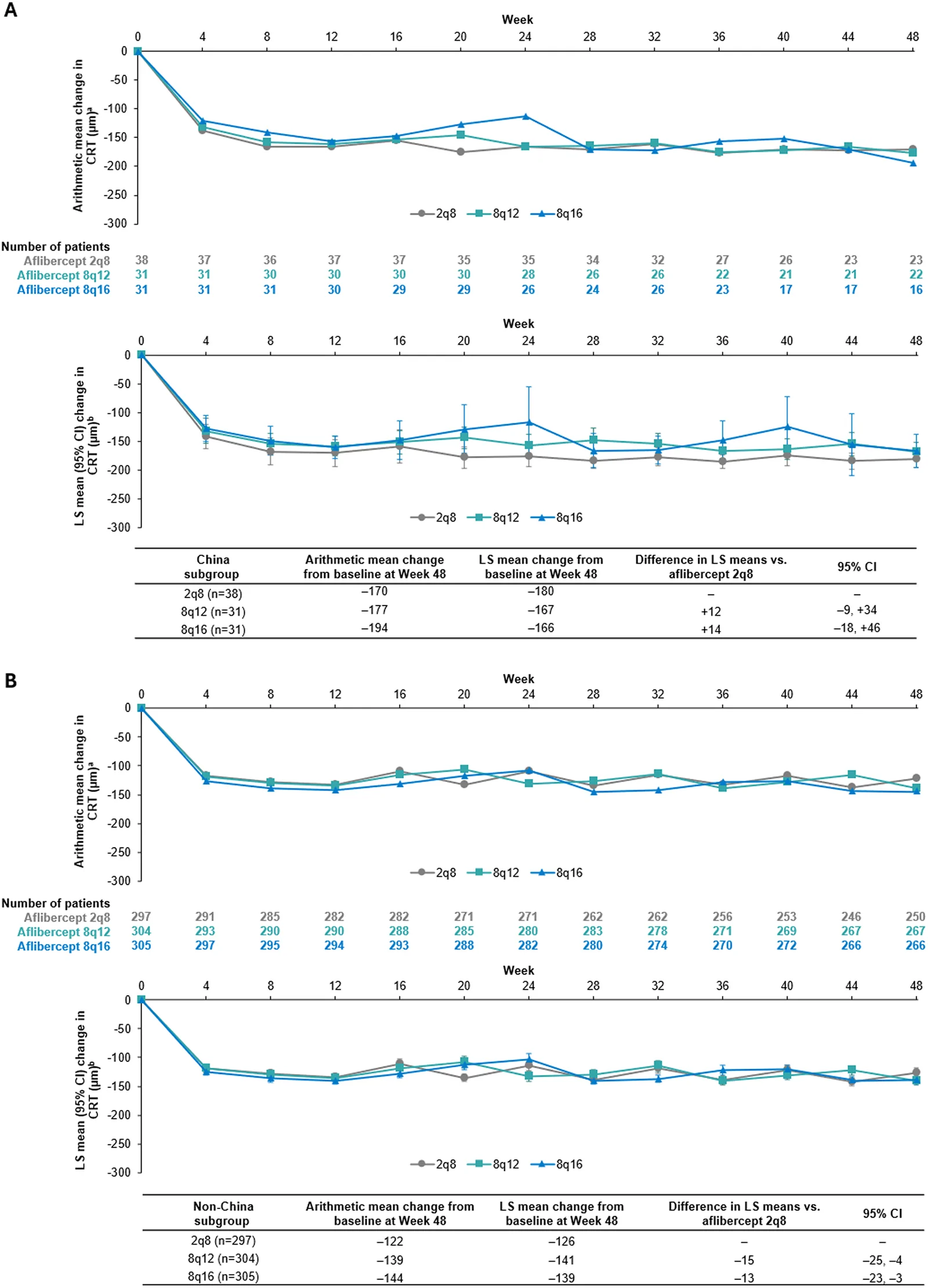
Arithmetic mean change (top panel) and LS mean change (bottom panel) in CRT through week 48 in the China (A) and non-China (B) subgroups. Full analysis set. aArithmetic mean changes based on OC prior to ICE. The number of patients included in the analysis, excluding patients with relevant intercurrent events, are provided for each timepoint. bLS mean data were generated using the MMRM, with baseline CRT as a covariate, treatment group (ie, aflibercept 2q8, 8q12, and 8q16), visit, and stratification variables (ie, geographical region [China vs the rest of the world] and baseline best-corrected visual acuity [ <60 vs ≥60]) as fixed factors, and terms for the interaction between baseline CRT and visit and the interaction between treatment and visit. 2q8, aflibercept 2 mg every 8 weeks; 8q12, aflibercept 8 mg every 12 weeks; 8q16, aflibercept 8 mg every 16 weeks. CI, confidence interval; CRT, central retinal thickness; ICE, intercurrent events; LS, least squares; MMRM, mixed model for repeated measures; OC, observed cases

Reductions in mean CRT were observed in both the China and non-China subgroups from baseline through week 48 (Fig. 3). Mean (SD) change from baseline in CRT at week 48 in the China subgroup was – 177 (144), – 194 (172), and – 170 (122) µm for patients in the 8q12, 8q16, and 2q8 groups, respectively. In this subgroup, the LS mean (95% CI) change in CRT from baseline at week 48 was − 167 (–183, −151), − 166 (–195, −137), and − 180 (–195, −165) µm for the 8q12, 8q16, and 2q8 groups, respectively (Fig. 3A).

In the non-China subgroup, the LS mean (95% CI) change in CRT from baseline at week 48 was − 141 (–148, −133), − 139 (–146, −133), and − 126 (–134, −118) µm for the 8q12, 8q16, and 2q8 groups, respectively (Fig. 3B).

### Durability

**Fig. 4:**
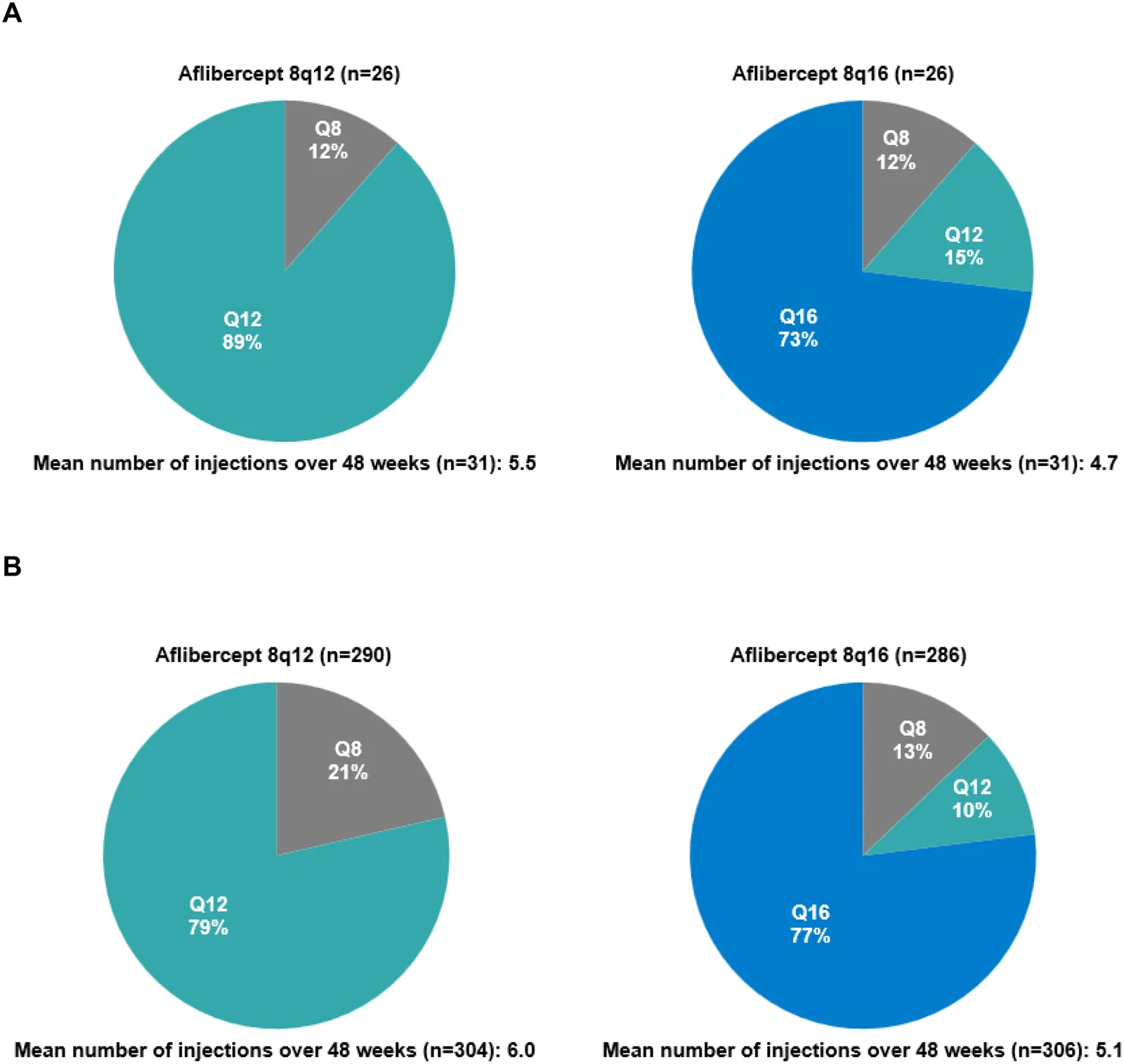
Proportion of patients in the 8q12 and 8q16 groups with shortened or maintained dosing intervals through week 48 in the China (**A**) and non-China (**B**) subgroups. Data are presented for patients who completed the week 48 visit. Q8, every 8 weeks; Q12, every 12 weeks; Q16, every 16 weeks; 8q12, aflibercept 8 mg every 12 weeks; 8q16, aflibercept 8 mg every 16 weeks

In the China and non-China subgroups, respectively, 89% and 83% of patients who received aflibercept 8 mg maintained ≥ 12-week dosing intervals through week 48. In the China 8q12 subgroup, 89% of patients maintained their assigned 12-week dosing interval through week 48. In the China 8q16 group, 73% of patients maintained their assigned 16-week dosing interval, and 89% maintained ≥ 12-week dosing intervals through week 48 (Fig. 4A). In the non-China 8q12 subgroup, 79% of patients maintained their assigned 12-week dosing intervals through week 48. In the non-China 8q16 subgroup, 77% of patients maintained their assigned 16-week dosing interval, and 87% maintained ≥ 12-week dosing intervals through week 48 (Fig. 4B).

### Safety

The proportion of patients with ocular TEAEs was 38.7% (12/31), 25.8% (8/31), and 38.5% (15/39) for the 8q12, 8q16, and 2q8 groups, respectively (Table 2). Ocular TEAEs reported in ≥ 5% of patients in any treatment group were cataract, dry eye, intraocular pressure increased, macular edema, retinal hemorrhage, vitreous floaters, and xerophthalmia (Supplementary Table 1). Serious ocular TEAEs were reported in 1 patient in the 8q12 group (cataract, choroidal detachment, and retinal detachment), 1 patient in the 8q16 group (skin laceration), and 1 patient in the 2q8 group (angle closure glaucoma).

In the non-China subgroup, the proportion of patients with ocular TEAEs was consistent across treatment groups (8q12: 38.5% [117/304]; 8q16: 38.8% [119/307]; 2q8: 38.7% [115/297], Table 2). The ocular TEAE reported in ≥ 5% of patients in any treatment group was reduced visual acuity (Supplementary Table 1). Serious ocular TEAEs were reported in 5 patients in the 8q12 group (2 reports of retinal detachment, 2 reports of intraocular pressure [IOP] increased, and 1 report of retinal hemorrhage), 4 patients in the 8q16 group (1 report each of angle closure glaucoma, retinal detachment, retinal hemorrhage, and vitreous hemorrhage), and 1 patient in the 2q8 group (retinal hemorrhage).

No cases of endophthalmitis, iritis, occlusive retinal vasculitis, vitritis, chorioretinitis, or iridocyclitis were reported in the China subgroup. In the non-China subgroup, single cases of vitreal cells, chorioretinitis, iritis, and vitritis were reported in the 8q12 group, iridocyclitis in the 8q16 group, and iridocyclitis and vitreal cells in the 2q8 group.

In the China subgroup, increased IOP was reported as a TEAE by the investigators in 3.2% (1/31), 9.7% (3/31), and 5.1% (2/39) of patients in the 8q12, 8q16, and 2q8 groups, respectively (Table 2). These cases were considered to be mild in severity by the study investigator. In the non-China subgroup, increased IOP was reported in 3.3% (10/304), 2.0% (6/307), and 1.7% (5/297) of patients in the 8q12, 8q16, and 2q8 groups, respectively (Table 2).

In the China subgroup, non-ocular TEAEs were observed in 51.6% (16/31) of patients in the 8q12 group, 41.9% (13/31) of patients in the 8q16 group, and 43.6% (17/39) of patients in the 2q8 group. Non-ocular TEAEs that were reported in ≥ 5% of patients in any treatment group were dyspepsia, nasopharyngitis, toothache, upper respiratory tract infection, back pain, and hypertension. Serious non-ocular TEAEs were reported in 16.1% (5/31), 12.9% (4/31) and 10.3% (4/39) of patients in the 8q12, 8q16 and 2q8 groups, respectively (Table 2). Overall, 14.5% (9/62) of patients who received aflibercept 8 mg experienced serious non-ocular TEAEs (Table 2). In the non-China subgroup, non-ocular TEAEs were observed in 52.3% (159/304), 55.0% (169/307), and 54.2% (161/297) of patients in the 8q12, 8q16, and 2q8 groups, respectively. Apart from COVID-19 infection (3.3% [10/304], 6.8% [21/307], and 3.7% [11/297] of patients in the 8q12, 8q16 and 2q8 groups, respectively), no other non-ocular TEAEs were reported in ≥ 5% of patients. Non-ocular serious TEAEs occurred in 9.5% (29/304), 9.1% (28/307), and 14.1% (42/297) of patients in the 8q12, 8q16 and 2q8 subgroups, respectively.

Overall, 9 deaths were reported in the PULSAR trial through week 48, and all 9 deaths were reported in the non-China subgroup (3 in the 8q12 group, 1 in the 8q16 group, and 5 in the 2q8 group). None of the deaths were considered to be related to aflibercept treatment. No new safety concerns were identified in the China and non-China subgroups.

**Table 2:** Summary of key adverse events in the China and non-China subgroups through week 48 of PULSAR

Adverse events, *n* (%)	China				Non-China			
	Aflibercept 2q8 (*n* = 39)	Aflibercept 8q12 (*n* = 31)	Aflibercept 8q16 (*n* = 31)	Combined aflibercept 8 mg (*n* = 62)	Aflibercept 2q8 (*n* = 297)	Aflibercept 8q12 (*n* = 304)	Aflibercept 8q16 (*n* = 307)	Combined aflibercept 8 mg (*n* = 611)
Ocular TEAE in the study eye								
Any ocular TEAE	15 (38.5)	12 (38.7)	8 (25.8)	20 (32.3)	115 (38.7)	117 (38.5)	119 (38.8)	236 (38.6)
Any serious ocular TEAE	1 (2.6)	1 (3.2)	1 (3.2)	2 (3.2)	1 (0.3)	5 (1.6)	4 (1.3)	9 (1.5)
Non-ocular TEAE								
Any non-ocular TEAE	17 (43.6)	16 (51.6)	13 (41.9)	29 (46.8)	161 (54.2)	159 (52.3)	169 (55.0)	328 (53.7)
Any serious non-ocular TEAE	4 (10.3)	5 (16.1)	4 (12.9)	9 (14.5)	42 (14.1)	29 (9.5)	28 (9.1)	57 (9.3)
Any TEAE of intraocular inflammation in the study eye								
Iridocyclitis	0	0	0	0	1 (0.3)	0	1 (0.3)	1 (0.2)
Vitreal cells	0	0	0	0	1 (0.3)	1 (0.3)	0	1 (0.2)
Chorioretinitis	0	0	0	0	0	1 (0.3)	0	1 (0.2)
Iritis	0	0	0	0	0	1 (0.3)	0	1 (0.2)
Vitritis	0	0	0	0	0	1 (0.3)	0	1 (0.2)
Intraocular pressure increased	2 (5.1)	1 (3.2)	3 (9.7)	4 (6.5)	5 (1.7)	10 (3.3)	6 (2.0)	16 (2.6)

2q8, aflibercept 2 mg every 8 weeks; 8q12, aflibercept 8 mg every 12 weeks; 8q16, aflibercept 8 mg every 16 weeks; TEAE, treatment-emergent adverse event

## Discussion

This subgroup analysis of PULSAR is the first to specifically evaluate the efficacy, durability, and safety of aflibercept 8 mg in patients with nAMD from China. At week 48, robust gains in BCVA ranging from + 9.7 to + 13.2 letters were observed in the China subgroup with aflibercept 8 mg and aflibercept 2 mg. Improvements in BCVA were also observed with aflibercept 8 mg and aflibercept 2 mg in the non-China subgroup, with gains, ranging from + 5.7 to + 7.0 letters, similar to those observed in the overall PULSAR population (BCVA gains ranged from + 5.9 to + 7.0 letters) [12].

Rapid fluid resolution was apparent at week 16 and was sustained through week 48 in all treatment groups in both China and non-China subgroups. Within each subgroup, patients across treatment groups had numerically similar visual and anatomic improvements over the 48 weeks, despite patients in the 8q12 and 8q16 groups receiving fewer injections than those in the 2q8 group, consistent with findings in the overall PULSAR population [12]. Numerically greater reductions in CRT were observed across treatment groups in the China subgroup (ranging from − 166 to − 180 μm) compared with the non-China subgroup (ranging from − 126 to − 141 μm).

Of the patients in the China subgroup who received aflibercept 8 mg and completed week 48, 89% maintained dosing intervals of ≥ 12 weeks through week 48, and 73% of those in the 8q16 group maintained dosing intervals of ≥ 16 weeks. The high proportion of patients maintaining extended dosing intervals demonstrates adequate disease control, with fewer injections, in this patient population. It is acknowledged that these findings are based on protocol-driven criteria and may not be reflective of routine clinical practice where treatment decisions are often influenced by socio-economic and patient-related factors, which may affect both dosing interval and treatment adherence [7, 18]. Nevertheless, these results may have important implications for treat-and-extend regimens, which are widely used in clinical practice [19]. The durability of other therapeutic agents for nAMD has previously been investigated in Chinese patients in the phase 3 TENAYA and LUCERNE trials of faricimab 6 mg up to 16 weeks vs. fixed dosing with aflibercept 2 mg every 8 weeks [20]. In a subgroup analysis of the LUCERNE trial [21], 87% and 67% of patients from China achieved ≥ 12- and 16-week dosing intervals with faricimab, respectively, at week 48. However, differences in trial enrollment criteria and study protocol preclude robust comparisons of results from TENAYA and LUCERNE with PULSAR.

In this analysis, although visual improvements were generally consistent across the aflibercept 8 mg and aflibercept 2 mg groups within the China and non-China subgroups, higher BCVA gains overall were reported in the China subgroup compared with the non-China subgroup. Differences in the baseline characteristics between these subgroups, including distribution of CNV type, higher incidence of PCV, younger age and overall lower BCVA in the China subgroup, may have contributed to this outcome. The higher prevalence of PCV in the China subgroup compared with the non-China subgroup may have contributed to the observed higher visual gains, as PCV has been shown to respond favorably to anti-VEGF therapy [22–25]. Larger numerical BCVA gains have been reported among patients with poorer baseline vision, a pattern regularly observed in pivotal anti-VEGF trials [26, 27], and likely reflecting both regression to the mean and a ceiling effect in those with higher starting BCVA values. Differences in routine clinical practice may have also influenced the treatment responsiveness observed in this analysis, with results from a recent registry study suggesting the association of lower treatment initiation rates with Asian race, which may have potentially contributed to poorer baseline vision at the time of the study start [28].

However, the differences in LS means (95% CI) change from baseline to week 48 in BCVA between the aflibercept 8 mg and aflibercept 2 mg groups were comparable in the China and the non-China subgroups (+ 3.2 [–2.0, +8.4] and − 1.3 [–3.3, +0.7] letters for aflibercept 8q12 vs. 2q8, and − 0.3 [–5.2, +4.7] and − 1.1 [–3.1, +0.8] letters for aflibercept 8q16 vs. 2q8, respectively), suggesting that the overall benefit of aflibercept 8 mg and aflibercept 2 mg was consistent within both subgroups in this analysis.

Through week 48, aflibercept 8 mg demonstrated a safety profile consistent with that of aflibercept 2 mg in the China subgroup; although these findings should be interpreted in the context of the limited sample size of this subgroup, we note that they are consistent with the safety results in the non-China subgroup and the overall PULSAR population. No cases of intraocular inflammation, including endophthalmitis or occlusive retinal vasculitis, were reported.

The aflibercept 8 mg formulation delivers a 4-fold higher molar dose of aflibercept than the aflibercept 2 mg formulation and is administered in a 70 µL injection volume, whereas the aflibercept 2 mg formulation is delivered in a 50 µL injection volume. Differences in anatomic characteristics of the eye, such as the shallower anterior chamber depth, have been reported among aging individuals in South China compared with other groups [29, 30]. Despite these considerations, no clinically relevant differences in IOP-related or intraocular inflammation-related TEAEs were observed with aflibercept 8 mg vs. aflibercept 2 mg in the China subgroup.

Limitations of this subgroup analysis include the small sample size of the China subgroup, and relatively short follow-up period. Due to asynchronous dosing after week 12, the potential additional efficacy benefits of the higher dose across the treatment groups cannot be fully evaluated after this timepoint. Although the data from the current analysis provide important insights regarding treatment response in the China subgroup to aflibercept 8 mg, follow-up analysis of the PULSAR study would be important to provide information on long-term outcomes. As with any *post hoc* analysis of subgroups, these results are exploratory and hypothesis-generating and further prospective studies are warranted to confirm these findings.

## Conclusions

In patients from China with nAMD, aflibercept 8 mg demonstrated efficacy and a safety profile consistent with that of aflibercept 2 mg, with no new safety concerns identified. Visual and anatomic improvements reported with aflibercept 8 mg were maintained over extended dosing intervals and with fewer injections than with aflibercept 2 mg. The results presented here are in line with those reported for the overall PULSAR population.

## Supplementary Information

Below is the link to the electronic supplementary material.

Supplementary Material 1

## Data Availability

Availability of the data underlying this publication will be determined later according to Bayer’s commitment to the European Federation of Pharmaceutical Industries and Associations/Pharmaceutical Research and Manufacturers of America (EFPIA/PhRMA) ‘Principles for responsible clinical trial data sharing.’ This pertains to the scope, time point, and process of data access. As such, Bayer commits to sharing upon request from qualified scientific and medical researchers, participant-level clinical trial data, study-level clinical trial data, and protocols from clinical trials in participants for medicines and indications approved in the United States (US) and European Union (EU) as necessary for conducting legitimate research. This applies to data on new medicines and indications that have been approved by the EU and US regulatory agencies on or after 1 January 2014. Interested researchers can use www.clinicalstudydatarequest.com to request access to anonymized participant-level data and supporting documents from clinical studies to conduct further research that can help to advance medical science or improve patient care. Information on the Bayer criteria for listing studies and other relevant information is provided in the study sponsor’s section of the portal. Data access will be granted to anonymized participant-level data, protocols, and clinical study reports after approval by an independent scientific review panel. Bayer is not involved in the decisions made by the independent review panel. Bayer will take all necessary measures to ensure that participant privacy is safeguarded.

## Abbreviations

2q8: Aflibercept 2 mg every 8 weeks after 3 initial monthly injections
8q12: Aflibercept 8 mg every 12 weeks after 3 initial monthly injections
8q16: Aflibercept 8 mg every 16 weeks after 3 initial monthly injections
BCVA: Best-corrected visual acuity
CI: Confidence interval
CRT: Central retinal thickness
DRM: Dose regimen modification
ETDRS: Early Treatment Diabetic Retinopathy Study
ICGA: Indocyanine green angiography
IOP: Intraocular pressure
LS: Least squares
nAMD: Neovascular age-related macular degeneration
PCV: Polypoidal choroidal vasculopathy
SAF: Safety analysis set
SD: Standard deviation
TEAEs: Treatment-emergent adverse events
VEGF: Vascular endothelial growth factor

## References

[1] Blindness GBD, Vision Impairment C, Vision Loss Expert Group of the Global Burden of Disease S. Causes of blindness and vision impairment in 2020 and trends over 30 years, and prevalence of avoidable blindness in relation to VISION 2020: the Right to Sight: an analysis for the Global Burden of Disease Study. Lancet Glob Health. 2021;9(2):e144–60. 33275949 10.1016/S2214-109X(20)30489-7 PMC7820391

[2] Organization WH. World report on vision. 2019.

[3] Wong WL, Su X, Li X, Cheung CM, Klein R, Cheng CY, et al. Global prevalence of age-related macular degeneration and disease burden projection for 2020 and 2040: a systematic review and meta-analysis. Lancet Glob Health. 2014;2(2):e106–16. 25104651 10.1016/S2214-109X(13)70145-1

[4] Song P, Du Y, Chan KY, Theodoratou E, Rudan I. The national and subnational prevalence and burden of age-related macular degeneration in China. J Glob Health. 2017;7(2):020703. 29302323 10.7189/jogh.07.020703 PMC5735777

[5] Chaikitmongkol V, Sagong M, Lai TYY, Tan GSW, Ngah NF, Ohji M, et al. Treat-and-Extend Regimens for the Management of Neovascular Age-related Macular Degeneration and Polypoidal Choroidal Vasculopathy: Consensus and Recommendations From the Asia-Pacific Vitreo-retina Society. Asia Pac J Ophthalmol (Phila). 2021;10(6):507–18. 34839342 10.1097/APO.0000000000000445 PMC8673847

[6] Fundus Disease Group of Chinese Ophthalmologist A. [Evidence-based guidelines for diagnosis and treatment of age-related macular degeneration in China (2023)]. Zhonghua Yan Ke Za Zhi. 2023;59(5):347–66. 37032564 10.3760/cma.j.cn112142-20221222-00649

[7] Holz FG, Figueroa MS, Bandello F, Yang Y, Ohji M, Dai H, et al. Ranibizumab treatment in treatment-naive neovascular age-related macular degeneration: Results from LUMINOUS, a global real-world study. Retina. 2020;40(9):1673–85. 31764612 10.1097/IAE.0000000000002670 PMC7447127

[8] Okada M, Mitchell P, Finger RP, Eldem B, Talks SJ, Hirst C, et al. Nonadherence or nonpersistence to intravitreal injection therapy for neovascular age-related macular degeneration: A mixed-methods systematic review. Ophthalmology. 2021;128(2):234–47. 32763265 10.1016/j.ophtha.2020.07.060 PMC7403101

[9] Daien V, Finger RP, Talks JS, Mitchell P, Wong TY, Sakamoto T, et al. Evolution of treatment paradigms in neovascular age-related macular degeneration: a review of real-world evidence. Br J Ophthalmol. 2021;105(11):1475–9. 33130553 10.1136/bjophthalmol-2020-317434 PMC8543219

[10] Ehlken C, Ziemssen F, Eter N, Lanzl I, Kaymak H, Lommatzsch A, et al. Systematic review: non-adherence and non-persistence in intravitreal treatment. Graefes Arch Clin Exp Ophthalmol. 2020;258(10):2077–90. 32572607 10.1007/s00417-020-04798-2 PMC7550304

[11] Spooner KL, Mhlanga CT, Hong TH, Broadhead GK, Chang AA. The burden of neovascular age-related macular degeneration: a patient’s perspective. Clin Ophthalmol. 2018;12:2483–91. 30584267 10.2147/OPTH.S185052 PMC6287411

[12] Lanzetta P, Korobelnik JF, Heier JS, Leal S, Holz FG, Clark WL, et al. Intravitreal aflibercept 8 mg in neovascular age-related macular degeneration (PULSAR): 48-week results from a randomised, double-masked, non-inferiority, phase 3 trial. Lancet. 2024;403(10432):1141–52. 38461841 10.1016/S0140-6736(24)00063-1

[13] New Eylea™ 8 mg approved in Japan. 2024. Available from: https://www.bayer.com/media/en-us/new-eylea-8-mg-approved-in-japan/. Accessed 10 October 2025.

[14] Eylea™ 8 mg approved in China for wet age-related macular degeneration 2025. Available from: https://www.bayer.com/media/en-us/eylea-8-mg-approved-in-china-for-wet-age-related-macular-degeneration/. Accessed 10 October 2025.

[15] EMA Aflibercept (Eylea) summary of product characteristics. Nov 2021, updated Jan 2023. Available from https://www.ema.europa.eu/en/documents/product-information/eylea-epar-product-information_en.pdf. Accessed July 2025.

[16] FDA. Eylea HD (aflibercept) Prescribing information, updated 8/2023. Available from: https://www.bayer.com/media/en-us/eylea-8-mg-approved-in-china-for-wet-age-related-macular-degeneration/. Accessed 10 October 2025.

[17] Foss AJE, Almeida D, Cheung CMG, Ogura Y, de Cock E, Empeslidis T. To Treat or Not to Treat? Resolving the Question of Subretinal and Intraretinal Fluid in Age-Related Macular Degeneration: A Narrative Review. Ophthalmol Ther. 2025;14(3):489–514. 39904844 10.1007/s40123-025-01093-3 PMC11825970

[18] Licht A, Mavris Y, Latz C, Schuster AK, Ponto KA, Mirshahi A. Lost to follow-up while undergoing intravitreal injection therapy: barriers to adherence in a real-world setting. Int J Retina Vitreous. 2026;12(1):39. 10.1186/s40942-026-00808-3 PMC12927251 41673898

[19] Teo KYC, Eldem B, Joussen A, Koh A, Korobelnik JF, Li X, et al. Treatment regimens for optimising outcomes in patients with neovascular age-related macular degeneration. Eye (Lond). 2025;39(5):860–9. 39379523 10.1038/s41433-024-03370-0 PMC11933311

[20] Heier JS, Khanani AM, Quezada Ruiz C, Basu K, Ferrone PJ, Brittain C, et al. Efficacy, durability, and safety of intravitreal faricimab up to every 16 weeks for neovascular age-related macular degeneration (TENAYA and LUCERNE): two randomised, double-masked, phase 3, non-inferiority trials. Lancet. 2022;399(10326):729–40. 35085502 10.1016/S0140-6736(22)00010-1

[21] Zou W, Jiang Q, Wang Y, Wei W, Sun X, Basu K, et al. Efficacy, durability and safety of faricimab for patients with neovascular age-related macular degeneration: 48-week results from the phase 3 LUCERNE China subpopulation. Asia Pac J Ophthalmol (Phila). 2025,14:100142. 10.1016/j.apjo.2025.100142 39818248

[22] Koh A, Lai TYY, Takahashi K, Wong TY, Chen LJ, Ruamviboonsuk P, et al. Efficacy and Safety of Ranibizumab With or Without Verteporfin Photodynamic Therapy for Polypoidal Choroidal Vasculopathy: A Randomized Clinical Trial. JAMA Ophthalmol. 2017;135(11):1206–13. 28983556 10.1001/jamaophthalmol.2017.4030 PMC5710379

[23] Lee WK, Iida T, Ogura Y, Chen SJ, Wong TY, Mitchell P, et al. Efficacy and safety of intravitreal aflibercept for polypoidal choroidal vasculopathy in the PLANET study: A randomized clinical trial. JAMA Ophthalmol. 2018;136(7):786–93. 29801063 10.1001/jamaophthalmol.2018.1804 PMC6136040

[24] Park JY, Park YJ, Park SJ, Park KH, Yeo JH, Kim JG, et al. Comparison of visual outcomes of polypoidal choroidal vasculopathy and typical neovascular age-related macular degeneration-up to 10 years of follow-up. Acta Ophthalmol. 2022;100(8):e1579–88. 35363434 10.1111/aos.15149

[25] Roh HC, Kim SJ, Kang SW, Eun JS, Choi KJ. Long-term outcomes of polypoidal choroidal vasculopathy in comparison with typical exudative age-related macular degeneration. Graefes Arch Clin Exp Ophthalmol. 2022;260(1):83–92. 34350467 10.1007/s00417-021-05190-4

[26] Schmidt-Erfurth U, Kaiser PK, Korobelnik JF, Brown DM, Chong V, Nguyen QD, et al. Intravitreal aflibercept injection for neovascular age-related macular degeneration: ninety-six-week results of the VIEW studies. Ophthalmology. 2014;121(1):193–201. 24084500 10.1016/j.ophtha.2013.08.011

[27] Brown DM, Kaiser PK, Michels M, Soubrane G, Heier JS, Kim RY, et al. Ranibizumab versus verteporfin for neovascular age-related macular degeneration. N Engl J Med. 2006;355(14):1432–44. 17021319 10.1056/NEJMoa062655

[28] Acharya B, Momenaei B, Zhang Q, Hyman L, Haller JA, Consortium IAC. Disparities in Presentation and Anti-VEGF Therapy Initiation for Neovascular Age-Related Macular Degeneration: An Analysis of the Academy IRIS(R) Registry (Intelligent Research in Sight). Ophthalmology. 2025;132(12):1411–21. 40738331 10.1016/j.ophtha.2025.07.024 PMC13448695

[29] He M, Huang W, Zheng Y, Alsbirk PH, Foster PJ. Anterior chamber depth in elderly Chinese: the Liwan eye study. Ophthalmology. 2008;115(8):1286. 18471885 10.1016/j.ophtha.2007.12.003

[30] Wang D, Qi M, He M, Wu L, Lin S. Ethnic difference of the anterior chamber area and volume and its association with angle width. Invest Ophthalmol Vis Sci. 2012;53(6):3139–44. 22531702 10.1167/iovs.12-9776 PMC3383186

