# Supplementary material for: Intravitreal aflibercept 8 mg in patients with neovascular age-related macular degeneration from China: 48-week results from the PULSAR trial
Source: Int J Retina Vitreous. 2026 Jun 26;12:123. doi: 10.1186/s40942-026-00876-5 (PMC13563726; doi:10.1186/s40942-026-00876-5)
Supplement: Supplementary file 1 [file 40942_2026_876_MOESM1_ESM.docx]

**Supplementary Information**

**Methods**

MMRM method included baseline BCVA as a covariate, and treatment group and stratification variables (baseline BCVA [<60 vs. ≥60 letters]) as fixed factors, as well as terms for the interaction between baseline BCVA and visit, and for the interaction between treatment and visit. Consistent with the hypothetical strategy of the primary estimand, data following the first occurrence of any relevant intercurrent events were excluded from the analysis and missing data were handled implicitly by the MMRM. A similar approach was used to evaluate the difference in LS mean change from baseline in CRT, using baseline CRT as a covariate and terms for the interaction between baseline CRT and visit.

**Supplementary Fig. 1** Trial profile of the China and non-China subgroups from the PULSAR trial.

Full analysis set. ^a^Please refer to Lanzetta et al., *Lancet* 2024 for more information [12]. 2q8, aflibercept 2 mg every 8 weeks; 8q12, aflibercept 8 mg every 12 weeks; 8q16, aflibercept 8 mg every 16 weeks; COVID-19, coronavirus disease 2019.

**Supplementary Table 1** Ocular TEAEs reported in ≥5% of patients in the China and non-China subgroups in the PULSAR trial

| TEAEs, n (%) | China | | | | Non-China | | | |
| --- | --- | --- | --- | --- | --- | --- | --- | --- |
|  | Aflibercept 2q8 (n=39) | Aflibercept 8q12 (n=31) | Aflibercept 8q16 (n=31) | Combined aflibercept  8 mg  (n=62) | Aflibercept  2q8  (n=297) | Aflibercept 8q12  (n=304) | Aflibercept 8q16  (n=307) | Combined aflibercept  8 mg  (n=611) |
| Cataract | 1 (2.6) | 2 (6.5) | 0 | 2 (3.2) | 9 (3.0) | 10 (3.3) | 12 (3.9) | 22 (3.6) |
| Dry eye | 1 (2.6) | 2 (6.5) | 0 | 2 (3.2) | 3 (1.0) | 3 (1.0) | 5 (1.6) | 8 (1.3) |
| Intraocular pressure increased | 2 (5.1) | 1 (3.2) | 3 (9.7) | 4 (6.5) | 5 (1.7) | 10 (3.3) | 6 (2.0) | 16 (2.6) |
| Macular edema | 2 (5.1) | 1 (3.2) | 0 | 1 (1.6) | 6 (2.0) | 0 | 7 (2.3) | 7 (1.1) |
| Reduced visual acuity | 0 | 0 | 0 | 0 | 20 (6.7) | 12 (3.9) | 18 (5.9) | 30 (4.9) |
| Retinal hemorrhage | 1 (2.6) | 2 (6.5) | 0 | 2 (3.2) | 13 (4.4) | 9 (3.0) | 10 (3.3) | 19 (3.1) |
| Vitreous floaters | 2 (5.1) | 0 | 0 | 0 | 9 (3.0) | 4 (1.3) | 12 (3.9) | 16 (2.6) |
| Xerophthalmia | 2 (5.1) | 1 (3.2) | 0 | 1 (1.6) | 0 | 0 | 0 | 0 |

Safety analysis set. 2q8, aflibercept 2 mg every 8 weeks; 8q12, aflibercept 8 mg every 12 weeks; 8q16, aflibercept 8 mg every 16 weeks; TEAE, treatment-emergent adverse event

**Supplementary Table 2** Independent Ethics Committees/Institutional Review Boards

| **Country** | **Name of Independent Ethics Committee/Institutional Review Board** |
| --- | --- |
| Argentina | J. E. Uriburu 774 Piso 1°, Buenos Aires, Buenos Aires 1027, ARGENTINA |
| Australia | Research Governance Unit, St Vincent's Hospital, 41 Victoria Parade, Fitzroy, Victoria 3065, AUSTRALIA |
| Austria | Medizinische Universität Graz, Ethik-Kommission, Auenbruggerplatz 2, 8036 Graz, AUSTRIA |
| Bulgaria | Ethics Committee for Clinical Trials, 8 Damyan Gruev Str, Sofia 1303, BULGARIA |
| Canada | Western University Research Ethic Committee, 1393 Western Road, Room 5150, Support Services Building, London, Ontario N6A 3K7, CANADA |
| Canada | Advarra, 6940 Columbia Gateway Drive, Suite 110, Columbia, Maryland 21046, UNITED STATES |
| Canada | The University of Manitoba Biomedical Research Ethics Board, Bannatyne Campus (address not provided in text) |
| China | Shanghai General Hospital Institutional Review Board, No. 100 Haining Road, Shanghai, China |
| China | Medical Ethics Committee of Union Hospital affiliated to Tongji Medical College of Huazhong University of Science and Technology, No. 1277 Jiefang Road, Wuhan, Hubei, China |
| China | Medical Ethics Committee of Henan Provincial Ophthalmology Hospital, No. 7 Weiwu Road, Jinshui District, Zhengzhou, Henan, China |
| China | Ethics Committee of The First Affiliated Hospital of Xinjiang Medical University, No. 137 South of Liyu Shan Road, Urumqi, Xinjiang, China |
| China | Ethical Committee for Clinical Trial Review of Tianjin Eye Hospital, No. 4 Guansu Road, Heping District, Tianjin, China |
| China | Beijing Hospital Ethics Committee, 1 Dahua Road, Dongcheng District, Beijing, China |
| China | The Medical Ethics Committee of The Second Hospital of Jilin University, No. 218 Ziqiang Street, Nanguan District, Changchun, Jilin, China |
| China | EC of Tianjin Medical University General Hospital, No. 154 Anshandao, Heping District, Tianjin, China |
| China | Ethics Committee of Huazhong University of Science and Technology, No. 1095 Jiefang Road, Qiaokou District, Wuhan, Hubei, China |
| China | Ethics Committee of Eye Hospital Affiliated to Wenzhou Medical University, No. 270 Xueyuan West Road, Wenzhou, Zhejiang, China |
| China | Ethics Committee of The People’s Hospital of Ningxia Hui Autonomous Region, No. 936 Huanghe East Road, Jinfeng District, Ningxia, China |
| China | Ethics Committee of Aier Eye Hospital, No. 191 Huanshizhong Road, Yuexiu District, Guangzhou, Guangdong, China |
| China | Medical Ethics Committee of The First Affiliated Hospital of Nanchang University, No. 17 Yong Wai Zheng Jie Street, Nanchang, Jiangxi, China |
| China | Ethics Committee of Beijing Aier Yingzhi Eye Hospital Co., Ltd, No. 12 Panjiayuan Nanli, Chaoyang District, Beijing, China |
| China | Aier Eye Hospital (Chengdu) Drug Clinical Trial Ethics Committee, No. 115 West First Section of Yihuan Road, Chengdu, Sichuan Province, China |
| China | Medical Ethics Committee of Jiangxi Provincial People’s Hospital, No. 266 Feng He Bei Road, Hong Gu Tan District, Nanchang, Jiangxi, China |
| China | The Medical Ethics Committee of Shenyang He Eye Hospital, No. 128 North Huanghe Street, Shenyang, Liaoning, China |
| China | Ethics Committee of Biomedical Study of Peking University First Hospital, No. 8 Xushiky Street, Xucheng District, Beijing, China |
| China | Ethics Committee of Zhejiang Provincial People’s Hospital, No. 158 Shangtang Road, Hangzhou, Zhejiang, China |
| China | Clinical Research Ethics Committee of the First Affiliated Hospital of Zhejiang University School of Medicine, No. 17 Old Zhejiang Dazhi Road (3rd floor, Building 2, University Road Campus), Hangzhou, Zhejiang, China |
| China | Clinical Trial Branch of Medical Ethics Committee of the First Affiliated Hospital of Fujian Medical University, No. 20 Chazhong Road, Taijiang District, Fuzhou, Fujian, China |
| China | The Clinical Trials Ethics Committee of Shenyang Aier Eye Optometry Hospital, No. 11 Weft Road, Heping District, Shenyang, Liaoning, China |
| China | Clinical Trial Ethics Committee of Chongqing Aier Ophthalmology Hospital, Nongken Building, No. 2 Huatang Road, Huaxin Street, Jiangbei District, Chongqing, China |
| China | Ethics Committee of Capital Medical University (CMU) – Beijing Tongren Hospital East – Beijing Tongren Eye Center, No. 1 Dongjiaomin Lane, Dongcheng District, Beijing, China |
| China | Ethics Review Committee of Peking University People’s Hospital, No. 11 Xizhimen Nan Street, Xicheng District, Beijing, China |
| China | Medical Ethics Committee of Tianjin Medical University Eye Hospital, No. 251 Fu Kang Road, Nankai District, Tianjin, China |
| China | Clinical Trial Ethics Committee of First Affiliated Hospital of Xi’an Jiaotong University, No. 277 West Yanta Road, Xi’an, Shaanxi, China |
| China | Drug Clinical Trial Ethics Committee of The Second Hospital of Anhui Medical University, No. 678 Furong Road, Jingjijishukaifa District, Hefei, Anhui, China |
| China | Medical Ethics Committee of Xuzhou First People’s Hospital, No. 269 Daxue Road, Tongshan District, Xuzhou, Jiangsu, China |
| China | Clinical Trial Ethics Committee of Hebei Eye Hospital, No. 399 Quanbeidongda Street, Xingtai, Hebei, China |
| China | Institutional Review Board of Shanghai Tongji Hospital, No. 389 Xincun Road, Putuo District, Shanghai, China |
| China | Drug Clinical Trial Ethics Committee of Tonghua Central Hospital, No. 176 Xinguang Road, Dongchang District, Tonghua, Jilin, China |
| China | Shijiazhuang People’s Hospital Drug Clinical Trial Ethics Committee, No. 365 Jianhua South Main Street, Yuhua District, Shijiazhuang, Hebei, China |
| Czech Republic | Eticka komise Axon Clinical, s.r.o, Sevce Matouse 433/26, Praha 4, 140 00, CZECH REPUBLIC |
| Czech Republic | Fakultni nemocnice Ostrava, 17. listopadu 1790, Ostrava–Poruba, Ostrava-město 708 52, CZECH REPUBLIC |
| Czech Republic | FN Hradec Kralove, Sokolska 581, Hradec Kralove 500 05, CZECH REPUBLIC |
| Czech Republic | FN Kralovske Vinohrady, Srobarova 50, Praha 10 100 34, CZECH REPUBLIC |
| Czech Republic | Lexum a.s., Antala Staska 1670/80, Praha 4, 140 00, CZECH REPUBLIC |
| Czech Republic | Fakultni nemocnice Plzen, Edvarda Benese 1128/13, Plzen 305 99, CZECH REPUBLIC |
| Czech Republic | Fakultni nemocnice Brno, Jihlavska 20, Brno 625 00, CZECH REPUBLIC |
| Estonia | Ethics committee for medicinal products, Nooruse 1, Tartu, ESTONIA |
| France | CPP SUD-Ouest et Outre-Mer II, 10 Chemin du raisin, Toulouse, Haute-Garonne, FRANCE |
| Georgia | EC of “Inova” LTD, 6 Z. Anjaparidze St (first lane), Tbilisi 0186, GEORGIA |
| Georgia | EC of Chichua Medical Center “Mzera”, Tsinandali St 9, Tbilisi 0144, GEORGIA |
| Georgia | EC of Caucasus Medical Centre, Kavtaradze Str. 23, Tbilisi 0186, GEORGIA |
| Georgia | EC of LTD “Clinic LJ”, 3/6a Sarajishvili–Chechelashvili, Kutaisi 4600, GEORGIA |
| Hungary | Medical Research Council Ethics Committee for Clinical Pharm., Alkotmány u. 25, 1054 Budapest, HUNGARY |
| Israel | Meir Medical Center, 59 Tshernichovsky St., Kfar-Saba, HaMerkaz 4428164, ISRAEL |
| Israel | The Chaim Sheba Medical Center, Ophthalmology Department, The Goldschleger Eye Institute Building, Tel-Hashomer 52621, ISRAEL |
| Israel | Assuta Hashalom, Yigal Allon St. 96, Tel-Aviv, HaMerkaz 6789140, ISRAEL |
| Israel | Helsinki Committee of Shamir Medical Center (Assaf Harofeh), P.O. Beer Yaacov, Zerifin 7030000, ISRAEL |
| Israel | Helsinki Committee of Barzilai Medical Center, 2 Hahistadrut St., Ashkelon 7830604, ISRAEL |
| Israel | Helsinki Committee of Carmel M, 7 Michal Street, Haifa, HaZafon 3436212, ISRAEL |
| Israel | Rambam Health Care Campus, 8 Haaliya Hashniya Street, Bat Galim (Old Building, Floor 4), Haifa 31096, ISRAEL |
| Israel | Kaplan Medical Center Helsinki, Hagalil Street, Rehovot, HaDarom 7610001, ISRAEL |
| Israel | Hadassah Medical Organization, Kiryat Hadassah, Jerusalem 91120, ISRAEL |
| Italy | Comitato Etico Unico Regionale, Piazza Ospedale 1, Trieste 34125, ITALY |
| Italy | Comitato Etico IRCCS Ospedale San Raffaele, Via Olgettina 60, Milano 20132, ITALY |
| Italy | Comitato Etico Indipendente presso la Fondazione Policlinico Tor Vergata di Roma, Viale Oxford 81, Roma 00133, ITALY |
| Italy | COMITATO ETICO CENTRALE IRCCS – Sezione I.F.O. – Fondazione Bietti, Via Elio Chianesi 53, Roma 00144, ITALY |
| Italy | CER Umbria – Comitato Etico Regionale Umbria presso CREO – Centro di Ricerca Emato-Oncologico – Ospedale S. Maria della Misericordia, Piazzale Menghini, Perugia 06156 (PG), ITALY |
| Italy | Comitato Etico Catania 1, Via S. Sofia 78, Catania 95125, ITALY |
| Italy | Comitato Etico Indipendente AOU di Cagliari, Via Ospedale 54, Cagliari 09124, ITALY |
| Italy | Comitato Etico della Fondazione Policlinico Universitario Agostino Gemelli IRCCS Università Cattolica del Sacro Cuore, Largo Agostino Gemelli 8, Roma 00168, ITALY |
| Italy | Comitato Etico Milano Area 1 – ASST SACCO FATEBENEFRATELLI, Via G.B. Grassi 74, Università di Milano, Milano 20157, ITALY |
| Japan | Kobe University Hospital – IRB, 7-5-2 Kusunoki-Cho, Chuo-Ku, Kobe, Hyogo 650-0017, JAPAN |
| Japan | Hyogo Prefectural Amagasaki General Medical Center, 2-17-77 Higashinaniwa-cho, Amagasaki, Hyogo 660-0892, JAPAN |
| Japan | Hayashi Eye Hospital, 4-23-35 Hakataekimae, Hakata-ku, Fukuoka-city, Fukuoka 812-0011, JAPAN |
| Japan | Kokura Memorial Hospital – IRB, 3-2-1 Asano, Kokurakita-ku, Kitakyushu-city, Fukuoka, JAPAN |
| Japan | Nagoya City University Hospital, 1 Kawasumi, Mizuho-cho, Mizuho-ku, Nagoya, Aichi 467-8602, JAPAN |
| Japan | Tohoku University Hospital, 1-1 Seiryocho, Aoba-ku, Sendai, Miyagi 980-8574, JAPAN |
| Japan | Nara Medical University Hospital, 840 Shijo-cho, Kashihara 634-8522, JAPAN |
| Japan | Okayama University Hospital, 2-5-1 Shikata-Cho, Kita-Ku, Okayama, Okayama 700-8558, JAPAN |
| Japan | University of the Ryukyus Hospital, 207 Uehara, Nishihara-cho, Nakagami-gun, Okinawa 903-0215, JAPAN |
| Japan | Joint IRB, Sanyo Kochi Bldg. 3F, 1-14 Minamikibo, Kochi, JAPAN |
| Japan | IRB of Shiga University of Medical Science Hospital, Seta Tsukinowa-cho, Otsu 520-2192, JAPAN |
| Japan | Mie University Hospital, 2-174 Edobashi, Tsu, Mie 514-8507, JAPAN |
| Japan | Akita University Hospital IRB, 44-2 Hasunuma, Hiroomote, Akita, Akita, JAPAN |
| Japan | Review Board of Human Rights and Ethics for Clinical Studies (IRB), 13-2 Ichibancho, Chiyoda-ku, Tokyo 102-0082, JAPAN |
| Japan | Nagoya University Hospital, 65 Tsurumai-cho, Showa-ku, Nagoya, Aichi 466-8560, JAPAN |
| Japan | Kansai Medical University Hospital, 2-3-1 Shin-machi, Hirakata, Osaka, JAPAN |
| Japan | Hiroshima University Hospital IRB, 1-2-3 Kasumi, Minami-ku, Hiroshima 734-8551, JAPAN |
| Japan | Yamaguchi University Hospital IRB, 1-1-1 Minamikogushi, Ube-shi 755-8505, JAPAN |
| Japan | Aomori Prefectural Central Hospital IRB, 2-1-1 Higashitsukurimichi, Aomori, Aomori, JAPAN |
| Japan | National Hospital Organization National Tokyo Medical Center, 2-5-1 Higashigaoka, Meguro, Tokyo, JAPAN |
| Japan | ShinAkasaka Clinical Laboratory, 1-6-13 Horidome-cho, Nihonbashi, Chuo-ku, Tokyo 103-0012, JAPAN |
| Japan | Kanazawa University Hospital, 13-1 Takaramachi, Kanazawa, Ishikawa 920-8641, JAPAN |
| Japan | Tokushima University Hospital IRB, 2-50-1 Kuramoto-Cho, Tokushima 770-8503, JAPAN |
| Japan | Oita University Hospital, 1-1 Idaigaoka, Hasama-machi, Yufu, Oita 879-5593, JAPAN |
| Japan | Kozawa Eye Hospital and Diabetes Center IRB, 246-6 Yoshizawacho, Mito, Ibaraki, JAPAN |
| Japan | IRB of Tohoku Medical and Pharmaceutical University Hospital, 1-12-1 Fukumuro, Miyagino-ku, Miyagi 983-8512, JAPAN |
| Japan | The University of Tokyo Hospital, 7-3-1 Hongo, Bunkyo-ku, Tokyo 113-8655, JAPAN |
| Japan | University of Yamanashi Hospital, 1110 Shimokato, Chuo, Yamanashi 409-3898, JAPAN |
| Japan | Juntendo University Urayasu Hospital, 2-1-1 Tomioka, Urayasu, Chiba, JAPAN |
| Japan | Shinshu University Hospital, 3-1-1 Asahi, Matsumoto, Nagano 390-8621, JAPAN |
| Japan | Kansai Medical University MC IRB, 10-15 Fumizonocho, Moriguchi, Osaka, JAPAN |
| Japan | Japanese Red Cross Nagasaki Genbaku Hospital, 3-15 Mori-machi, Nagasaki 852-8511, JAPAN |
| Japan | Kanazawa Medical University Hospital, 1-1 Daigaku, Uchinada, Ishikawa 920-0293, JAPAN |
| Japan | Jimbo Orthopedics IRB, 5-38-41 Honcho, Koganei, Tokyo 184-0004, JAPAN |
| Japan | Osaka Metropolitan Univ Hospital, 1-5-7 Asahimachi, Abeno-ku, Osaka 545-8586, JAPAN |
| Japan | Sumitomo Hospital – IRB, 5-3-20 Nakanoshima, Kita-ku, Osaka City, Osaka 530-0005, JAPAN |
| Japan | Kindai University Hospital – IRB, 377-2 Ohno-higashi, Osaka-sayama 589-8511, JAPAN |
| Japan | Aichi Medical University Hospital, 1-1 Yazako-karimata, Nagakute, Aichi 480-1195, JAPAN |
| Japan | Japanese Red Cross Saitama Hospital IRB, 1-5 Shintoshin, Chuo-ku, Saitama, Saitama, JAPAN |
| Japan | University of Miyazaki Hospital, 5200 Kihara, Kiyotakecho, Miyazaki, Miyazaki 889-1692, JAPAN |
| Japan | St. Luke’s International Hospital IRB, 9-1 Akashi-cho, Chuo-ku, Tokyo, JAPAN |
| Japan | Keio University Hospital – Ophthalmology, 35 Shinanomachi, Shinjuku-ku, Tokyo 160-8582, JAPAN |
| Japan | Wakayama MC – IRB, 4-20 Komatsubara-dori, Wakayama City, Wakayama 640-8558, JAPAN |
| Japan | Kagawa University Hospital, 1750 |
